# Post-disaster food insecurity: Hurricane Maria as a case study

**DOI:** 10.1016/j.joclim.2024.100363

**Published:** 2024-11-24

**Authors:** Jill Mark, David de Angel Sola, Nicolas Rosario-Matos, Leyao Wang

**Affiliations:** a Division of Allergy and Immunology, Department of Medicine, Washington University School of Medicine, St. Louis, MO, USA; b San Juan City Hospital Department of Pediatrics, San Juan, Puerto Rico; c San Juan City Hospital Research Unit, San Juan, Puerto Rico; d Department of Biostatistics and Epidemiology, School of Public Health and Health Sciences, University of Massachusetts, Amherst, MA, USA; e Institute for Applied Life Sciences, University of Massachusetts, Amherst, MA, USA

**Keywords:** Climate change, Extreme weather, Hurricane, Disaster, Food insecurity, Nutrition, Health

## Abstract

**Background:**

Food insecurity is traditionally defined as a chronic condition linked to insufficient income, but in post-disaster contexts the condition may differ significantly, often with sudden onset and temporary nature. There is no specific definition for post-disaster food insecurity, making accurate data collection and development of effective interventions difficult.

**Methods:**

To explore this issue, we performed a pilot survey study (*n* = 22) in Puerto Rico after Hurricane Maria to evaluate food insecurity status, duration, and causes.

**Results:**

Over half of respondents self-reported experiencing food insecurity following Hurricane Maria. In general, food insecure respondents experienced more specific food group shortages that lasted longer than food secure respondents. The duration of food shortages significantly influenced participants' perceptions of food insecurity. The primary causes of food insecurity were identified as grocery store closures, a lack of food in stores, or other reasons not listed in the survey. These findings support what is known about the post-disaster setting with supply chain issues being a prominent mechanism of food insecurity as well as there being multiple mechanisms difficult to describe due to the complexity of the situation.

**Conclusions:**

Results highlight the need for refined definitions and assessments of post-disaster food insecurity that account for the temporal aspects of food shortages and the complexity of post-disaster settings. As climate change exacerbates the frequency and severity of natural disasters, addressing the nuances of post-disaster food insecurity is increasingly urgent to mitigate associated health risks.

## 1 Introduction

Food insecurity is a prominent public health issue. In 2022, the Food and Agriculture Organization of the United Nations (FAO) estimated 29.6 % of the global population were food insecure and lacked access to adequate food. There is a general assumption that food insecurity is a chronic condition resulting from a lack of income to purchase food [1,2]. The FAO states that “A person is food insecure when they lack regular access to enough safe and nutritious food for normal growth and development and an active and healthy lifestyle. This may be due to unavailability of food and/or lack of resources to obtain food. Food insecurity can be experienced at different levels of severity [3].” The United States Department of Agriculture (USDA) has a similar definition based on quality and quantity of food with an emphasis on long-term eating patterns [4].

However, post-disaster food insecurity tends to have a more sudden onset and is sometimes temporary, making the length of food shortages essential in defining the condition. Moreover, the field remains exploratory without a recognized definition from major organizations. One study raised a Disaster Food Security Framework based on pillars of availability, accessibility, acceptability, and agency over food before, during, and after a disaster [5], but they did not consider the length of food insecurity status after a disaster.

Food insecurity has been found to be associated with poor health outcomes. In adults, food insecurity has been linked to type 2 diabetes and cardiovascular disease with worse health exams overall [6,7]. For children, a lack of proper nutrient intake can lead to impaired development and cognitive functioning [8]. Additionally, infants who experienced prenatal food insecurity have been found to have altered gut microbiomes, putting them at risk for future poor health outcomes [9].

Climate change is increasing the rate and severity of natural disasters such as droughts, wildfires, floods, and hurricanes [[10], [11], [12]], leading to more people facing post-disaster food insecurity [13]. Therefore, defining post-disaster food insecurity is urgent and further studies of this problem are necessary to develop a standard for classifying food insecure residents after a disaster. This will allow organizations to equitably distribute aid.

## 2 Case presentation

To fill this knowledge gap, we conducted a small-scale survey study after Hurricane Maria, a category 4 storm, hit Puerto Rico on September 20, 2017. Maria caused severe damage estimated to cost $90 billion with a death toll greater than 5,000 [14,15]. Residents experienced severe trauma and multiple hardships including prolonged loss of power and food insecurity [16]. Specifically, severe crop destruction forced reliance on imports and damage made food distribution difficult, making food insecurity a prominent issue [17]. This study examined residents’ self-evaluation of food insecurity and importantly, the duration of food shortages by category. Results highlight the need to include the period of food insecurity in a post-disaster setting in defining the condition.

## 3 Methods

### 3.1 Study design

An online self-access questionnaire-based survey study was performed utilizing the RED Cap system to examine food insecurity after Hurricane Maria (IRB00002788). The Spanish survey was shared via email with residents identified from the San Juan Research Unit database who had participated in previous studies and were living in Puerto Rico during the six months following Hurricane Maria.

### 3.2 Measures and analysis

Demographic information collected included participants’ municipality and age. Participants were asked about general hardships such as lack of access to food, water, electricity and displacement. Individuals who reported food access issues were asked whether the quality, quantity, or both was affected. This subset of participants was also given a multiple-select question asking about the reasons for their food insecurity with choices of income loss, no transportation, local grocery store closed, no certain food supply in store, and other.

For a more precise investigation, all survey participants were asked whether they faced food shortages for specific food groups (fruits, vegetables, grains, protein, dairy). For all hardships and shortages reported, participants were asked for the duration of the condition.

Analysis of survey data was done at Washington University in St. Louis. All figures were created using Esri's ArcGIS Online web platform and Microsoft Excel.

## 4 Results

### 4.1 Post Hurricane Maria hardships

**Fig. 1:**
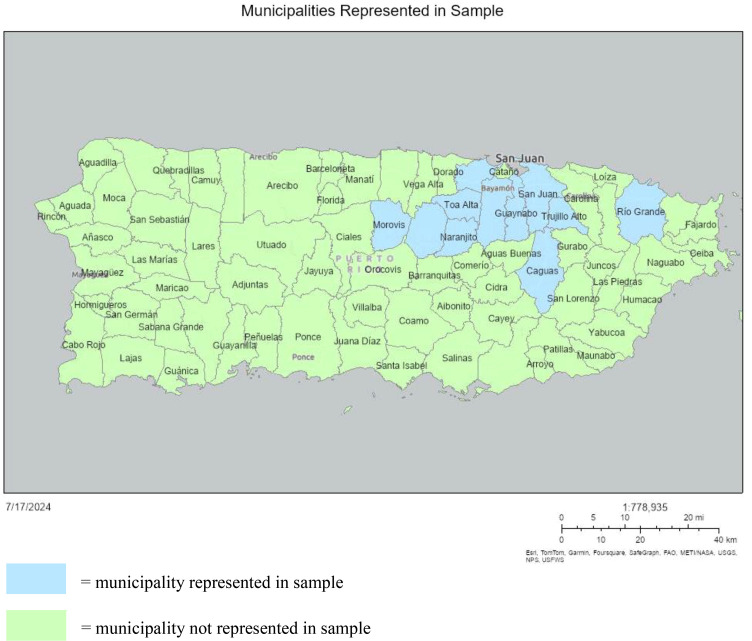
A map of municipalities in Puerto Rico categorized by whether the municipality was represented in our sample or not. Our sample was primarily concentrated in San Juan and the surrounding municipalities.

Twenty-two respondents from eleven different municipalities completed the online survey. Of respondents, 10 were from San Juan while most other from surrounding municipalities (Fig. 1). As shown in Table 1, most participants lost electricity which is consistent with data from Hurricane Maria and indicates respondents may well represent the hurricane-exposed population [14]. Thirteen participants lacked drinking water, while the same number claimed experiencing food insecurity. Five participants were displaced. For participants that experienced food insecurity, over half said both the quality and quantity of food was affected with four reporting only food quantity was affected and two reporting only food quality was affected.

**Table 1:** A table representing how many participants experienced certain hardships after Hurricane Maria including food insecurity, lack of drinking water, loss of electricity, and displacement. Twenty-two participants answered these questions.

Survey question asking about hardships due to Hurricane Maria	Number of subjects that answered Yes to the question (*n* = 22)
Did your household experience limited or no access to nourishment due to Hurricane Maria?	13
Did your household experience limited or no access to drinking water due to Hurricane Maria?	13
Did your household experience no electricity due to Hurricane Maria?	21
Was your household displaced due to Hurricane Maria?	5

### 4.2 Specific food group shortages

**Fig. 2:**
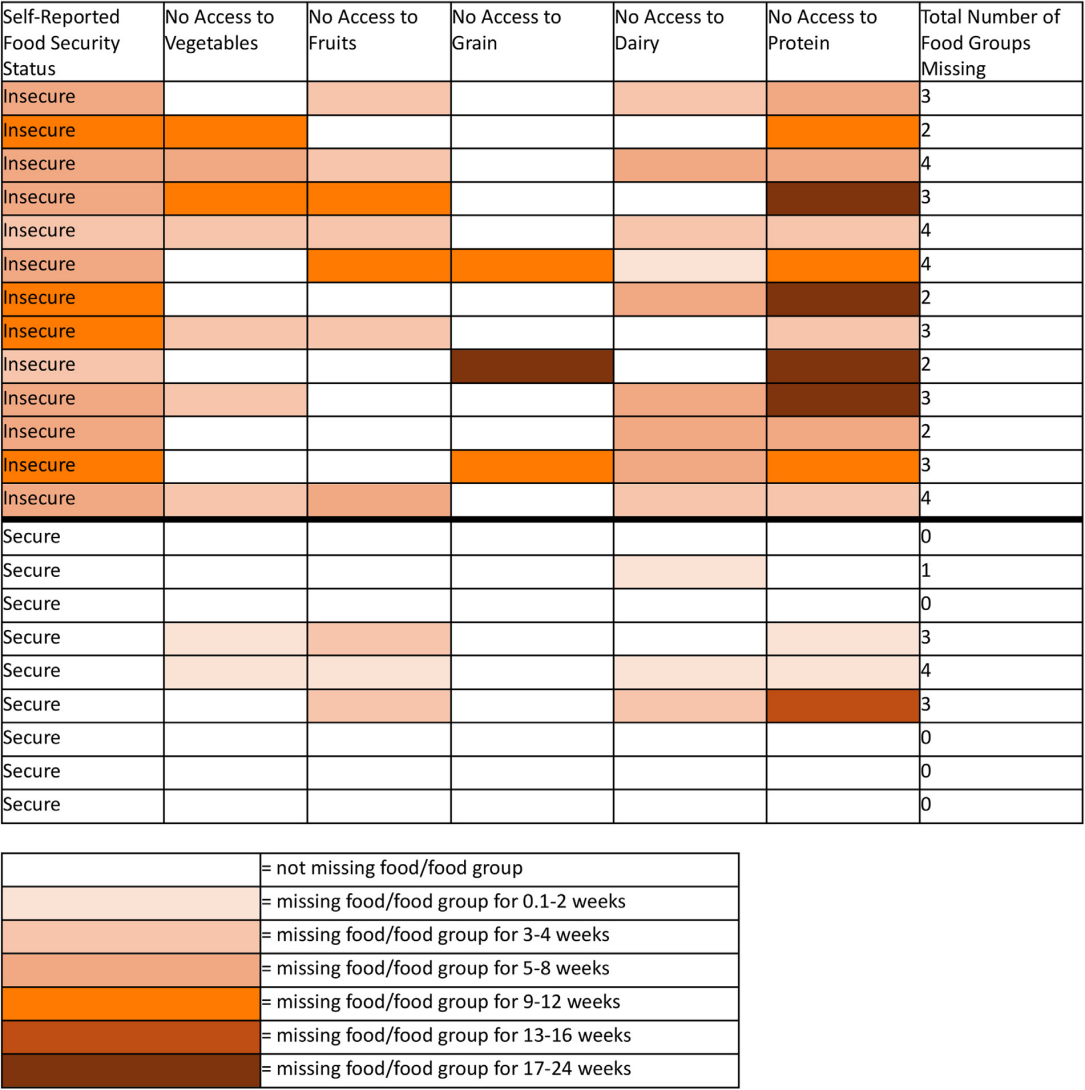
A table representing how long each participant experienced specific food group shortages with participants separated by their self-reported food security status. This status was determined by the respondents' answer to the question from Table 1 of "Did your household experience limited or no access to nourishment after Hurricane Maria?" with participants who answered "Yes" categorized as food insecure and those that answered "No" categorized as food secure. In general, food secure respondents still experienced food shortages but for shorter periods of time than the food insecure respondents.

All respondents were asked about whether they lacked access to the five key food groups, as determined by the USDA as necessary for health eating [18] (Fig. 2). For all food groups, a higher proportion of food insecure respondents were missing the specific food group versus food secure respondents. Protein was missing by all 13 of the food insecure respondents and three of the nine food secure respondents. Dairy was missing for nine respondents of the food insecure group and three respondents of the food secure group. Fruits were missing for seven of the food insecure and a three of food secure respondents. Vegetables were missing for seven of the food insecure respondents and two of the food secure respondents. Finally, grains were only missing for food insecure participants, with three respondents experiencing this shortage. Most participants correctly categorized themselves as food secure or food insecure based on their specific food shortages. The primary difference between food shortages for the food insecure and secure respondents was the duration of the shortage. In general, across all food groups, the length of the shortage was longer for the food insecure group compared to the food secure group.

### 4.3 Reasons for post-Hurricane food insecurity

**Fig. 3:**
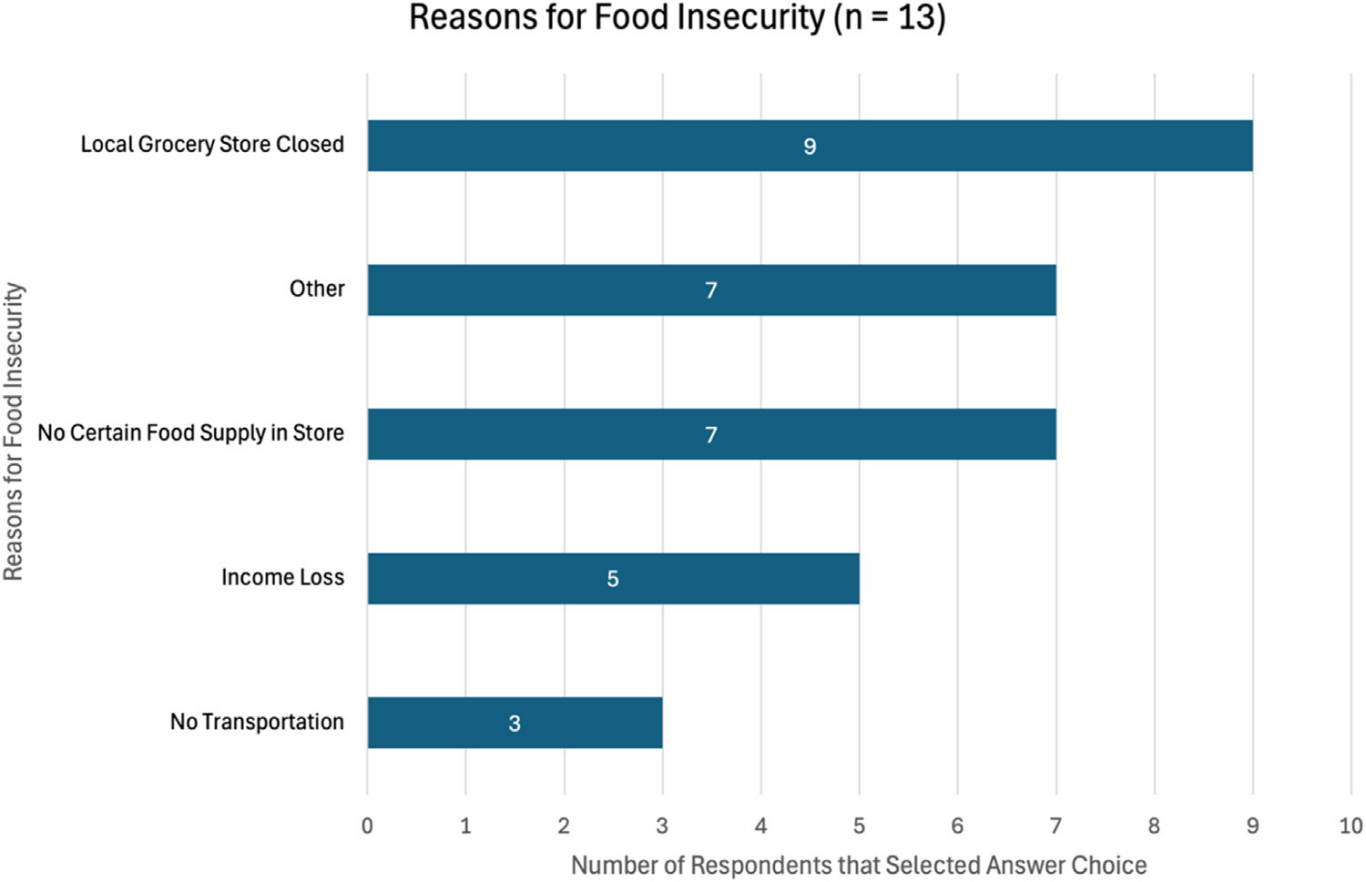
Reasons for Food Insecurity. A graph showing how many of the food insecure respondents selected each answer choice on a multiple-select question about the reasons for their food insecurity. Supply chain issues and a decreased presence of food in general appear to be prominent reasons for food insecurity with two of the highest reported reasons being related to grocery store closures and stock issues. Additionally, a high proportion of respondents selected "Other" indicating the presence of many other contributing factors to food insecurity after Hurricane Maria.

The food insecure group was queried about the causes of their food insecurity (Fig. 3). The most common reason was the closure of a local grocery store (nine respondents), followed by no certain food supply in store and other unlisted reasons (seven respondents each). The least reported reasons for food insecurity were income loss (five respondents) and no transportation (three respondents). Results indicate food insecurity was primarily due to supply chain disruptions. Additionally, the high proportion of respondents that reported having other reasons emphasizes how the post-disaster setting creates many contributing factors to food insecurity.

## 5 Discussion

Recognized FAO and USDA questionnaires assume food insecurity is chronic and primarily the result of a lack of income [1,2] but there are minimal tools specifically for post-disaster food insecurity. Our small-scale survey allowed us to examine food insecurity through close observation of each respondent and draw connections in their responses. The most significant finding was that the duration of food shortages appeared to be a key factor for respondents’ self-assessment of food insecurity – briefer shortages led to fewer respondents considering themselves food insecure, indicating time is an important factor. Additionally, the reported causes for food insecurity follow current understandings of supply chain problems contributing to post-disaster food insecurity [5] while also emphasizing the complexity of the condition.

The duration of food shortages should be explored in defining post-disaster food insecurity. Although some respondents initially reported having access to nourishment after Hurricane Maria (food secure group), they later indicated lacking access to specific food groups (Fig. 2). However, in general, these respondents were missing those food groups for less time than the food insecure group. The pattern indicates time is a key factor in the experience of food insecurity and whether people would classify their situation as severe enough to be considered food insecurity. Further research must assess the health impacts of brief food insecurity. This knowledge is essential in defining food insecure populations in a post-disaster setting and prioritizing aid distribution.

The sample shows physical barriers, such as grocery store closures and stock shortages are key factors of post-disaster food insecurity, aligning with the Disaster Food Security Framework [5]. These issues emphasize the condition can primarily be attributed to supply chain problems and infrastructure destruction.

Additionally, half of the food insecure respondents selected “Other” as a reason for their food insecurity, with all but one of these respondents also selecting answer choices listed. These responses provide evidence for post-disaster food insecurity as a complicated condition with mechanisms difficult to encompass in a multiple-choice question.

While these patterns provide insight into post-disaster food insecurity, analysis on the extent to which Hurricane Maria led to food insecurity is limited. Our survey did not assess participants’ food security status prior to Hurricane Maria to act as a baseline for the presence of post-disaster food insecurity. Puerto Rico had high rates of food insecurity prior to Hurricane Maria at 30–60 % (30 % were ineligible for federal food assistance who were food insecure) compared to 11.8 % in the U.S. overall [19]. Thus, a pre-post comparison is important. Additionally, disadvantaged groups that are more likely to experience chronic food insecurity are also expected to experience more adverse effects from natural disasters [20]. Combining these factors increases the likelihood of the coexistence of chronic food insecurity and disaster-related food insecurity. Moreover, by differentiating chronic and disaster-related food insecurity in questionnaires, we can better understand the specific mechanisms of post-disaster food insecurity.

## 6 Limitations

Aspects of this study limit the conclusions that can be drawn. First, the sample size is relatively small (*n* = 22), so the data may not represent trends on a broader scale. Additionally, the sample only represents the region of Puerto Rico surrounding San Juan. Since the impact of the storm may have been dependent on geographic location, the trends in this sample might not apply to others.

Another limitation is recall bias as respondents filled out the survey two years after the disaster. Especially for questions involving specific food groups or time frames of experiences, caution should be taken as the responses may not be completely accurate.

Voluntary bias may also be present as invitees that experienced food insecurity after Hurricane Maria could have been more likely to fill out the survey.

## 7 Conclusions

Given the impact of natural disasters on food insecurity and its health consequences, it is important for post-disaster food insecurity to be better defined and evaluated. As climate change increases the rate and severity of natural disasters [[10], [11], [12]], food insecurity becomes a more pressing matter. Our study showed that the duration of food shortages after Hurricane Maria impacted whether participants considered themselves food insecure, suggesting that the temporary nature of some cases should be reflected in its definition.

While this small-scale study does not completely encompass the post-disaster food insecurity experience, it contributes to broader research that supports the inclusion of time in its definition. It is also important to recognize that a singular definition of post-disaster food insecurity will be difficult to develop as each disaster poses unique challenges. However, small studies like this highlight aspects of the experience that can be considered by researchers to determine if it is relevant to their particular disaster study or post-disaster food insecurity research on a broader scale.

## Author agreement

All authors have seen and approved the final version of the manuscript. The article is the authors’ original work, has not received prior publication, and is not under consideration for publication elsewhere.

## CRediT authorship contribution statement

**Jill Mark:** Writing – review & editing, Writing – original draft, Formal analysis. **David de Angel Sola:** Writing – review & editing, Investigation, Data curation, Conceptualization. **Nicolas Rosario-Matos:** Writing – review & editing, Data curation, Project administration, Investigation, Conceptualization. **Leyao Wang:** Writing – review & editing, Project administration, Conceptualization.

## Declaration of competing interest

The authors declare that they have no known competing financial interests or personal relationships that could have appeared to influence the work reported in this paper.
